# A Smart Pillow for Health Sensing System Based on Temperature and Humidity Sensors

**DOI:** 10.3390/s18113664

**Published:** 2018-10-29

**Authors:** Songsheng Li, Christopher Chiu

**Affiliations:** 1 Department of Computer Engineering, Guangdong College of Business and Technology, Zhaoqing 526020, China; 2 Faculty of Engineering and Information Technology, University of Technologies, Sydney, Ultimo NSW 2007, Australia; christopher.chiu@uts.edu.au

**Keywords:** smart pillow, health sensing system, temperature, humidity, sensor

## Abstract

The quality of sleep affects the patient’s health, along with the observation of vital life signs such as body temperature and sweat in sleep, is essential in the monitoring of sleep as well as clinical diagnosis. However, traditional methods in recording physiological change amidst sleep is difficult without being intrusive. The smart pillow is developed to provide a relatively easy way to observe one’s sleep condition, employing temperature and humidity sensors by implanting them inside the pillow in strategic positions. With the patient’s head on the pillow, the roles of sensors are identified as main, auxiliary or environmental temperature, based on the differences of value from three temperature sensors, thus the pattern of sleep can be extracted by statistical analysis, and the body temperature is inferred by a specially designed Fuzzy Logic System if the head-on position is stable for more than 15 min. Night sweat is reported on data from the humidity sensor. Therefore, a cloud-based health-sensing system is built in the smart pillow to collect and analyze data. Experiments from various individuals prove that statistical and inferred results reflect normal and abnormal conditions of sleep accurately. The daily sleeping information of patients from the pillow is helpful in the decision-making of diagnoses and treatment, and users can change their habits of sleep gradually by observing the data with their health professional.

## 1. Introduction

With the advent of a globally aging society, the demand for remote health management systems is gaining acceptance within the medical community. The popularity of sensors, networks and big-data analytics has established a sound foundation for a networked health system. The problem that needs improvement is how one can observe physiological signs in a non-invasive and unobtrusive manner. One third of a person’s life is spent on sleep, therefore the quality and habit of sleep affects health, and the negative impacts of too much, too little, and poor-quality sleep are taken seriously. Physiological change in the middle of sleep is a significant indicator of health but is difficult to be observed using traditional methods. A pillow is used as a head support for comfort when sleeping. Hence, if data can be collected from a pillow under the no-perception condition, it will be a direct, effective, non-invasive and unperturbed way to analyze sleep and health. This article concentrates on the monitoring of body temperature, which is one of the most important physiological signs required for clinical diagnosis.

This paper provides a straight-forward way to observe sleep conditions by employing temperature and humidity sensors implanted inside the pillow. Body temperature will be measured in a way analogous to zero heat flux (ZHF) first, then compensated by a Fuzzy Logic system. Sensing data will be reported to the agent or mobile phone by Bluetooth Low Energy (BLE), then uploaded to cloud server via the Internet. Data analysis is then processed in the cloud and results presented via mobile application or web browser. The whole process and parties involved are collectively named the Health Sensing System. Experiments from various individuals prove that statistical and inferred results reflect normal and abnormal conditions of sleep accurately.

In the following of the paper, related works such as various measurements of body temperature, wearable instruments, transmission by BLE or ZigBee, and health monitoring system are presented first, then from the whole to the parts, details of the proposed health sensing system, smart pillow and how body temperature being extracted are explained gradually, and result of daily sleeping is provided and analyzed, which is followed by discussions, conclusions, and the idea of further research.

## 2. Related Works

In this section, the publications related to measurement of body temperature, wearable instruments and health monitoring systems are shown. 

The importance of body temperature has been agreed by researchers [1,2,3]; studies on the relationship between alertness, performance and body temperature prove performance and alertness is improved if the body temperature is increased, whether or not being synchronized with the internal biological clock [1]. Research from Lack et al. showed that insomnia in different phases of sleep is associated with abnormal body temperature rhythms [2] and treatments could adjust the circadian rhythm by bright light therapy. Coyne et al. found that the mortality from acute stroke was lower, and thus better outcomes in patients with mild hypothermia on admission, but actually worse if in a state of hyperthermia [3].

Information of body temperature has become indispensable, that researchers have dedicated their time to find unobtrusive and accurate measurement approaches. Use of the Kalman Filter is extensively adopted in engineering tracking problems [4], with a model developed to estimate the time course of core temperature (CT), employing a series of heart rate (HR) measurements as a main indicator. The filter is comprised of two models: a time update modeling how CT changes with uncertainty and an observation model mapping from HR to CT with uncertainty; then the filter is trained and validated. Then it is examined by the Bland–Altman limits of agreement (LoA) method, with the results accurate to provide practical indication of CT in the workplace, but it is not a replacement of direct measurement. Fox and Solman presented a new method for monitoring deep body temperature from the skin surface [5,6], known as ZHF, by creating a zone of zero heat-flow across the body shell to bring the deep body temperature to the skin surface, where it can be measured with a simple electronic thermometer. This method is proven to track CT very well in various ambient conditions [7,8] but using a heater to stop surface convection is an inherent weakness. An alternative method called dual-heat-flux (DHF) achieves similar outcomes by creating dual heat flux channels with less energy consumption and more sensors [9]. Huang et al. proposed improvements for DHF, such as modifying the probe size, measurement depth beneath the skin, and an aluminum cover to boost measurement accuracy [10,11], but also suggested the probe height be reduced by half.

A simpler device called a Double Sensor is invented based on skin temperature and heat flow, but without the use of a heating element [12]. This device employs only two temperature sensors, with the accuracy and precision being competitive with rectal and distal esophageal measurements [13,14]. Sim et al. embedded one DHF and two Double Sensors into a neck pillow to estimate core body temperature in the bed [15]. By this way, measurement can be derived from 3 different sleep positions but sleeping with a neck pillow is inconvenient. A garment made by Temperature Sensing Fabric (TSF) is manufactured on a knitting machine by laying fine metal wires into the double-layer knitted structure [16], which employs the principle that the metal wire changes its electrical resistance with the change in temperature. It is wearable and user-friendly, but difficult to produce in batches.

Wearable devices are increasingly popular because of the advancement of the electronics industry, with technologies making low-cost, comfortable, and unobtrusive devices possible [17]. For instance, high-sensitivity and low-cost printed wearable temperature sensors were developed [18,19], which is synthesized by poly (3,4-ethlenedioxythiophene) poly (styrene sulfonate) (PEDOT: PSS) and carbon nanotubes (CNTs). This solves the issue of size, although the next issue to be addressed is the method of low-power wireless transmission for uploading measurements; hence BLE being the main candidate.

A ring-shaped wrap for the finger was developed by Miah et al. [20], which integrates infrared Rx and Tx for the heart rate and temperature sensor. The sensing data is transferred to Android mobile in real time by BLE, then any abnormal signs can trigger an alarm, but the device is still in its experimental stages. A forehead-worn thermometer that connects to mobile via Bluetooth is presented [21], which is based on multiple Artificial Neural Networks (ANNs) in which certain hidden neurons are adaptively selected according to a combination of temperatures, but balancing estimation accuracy, computational load, and delay time needs to be addressed. Boano et al. designed and built a non-invasive wearable wireless monitoring prototype for accurate body temperature measurements, that is accurate to 0.02 °C and provides real-time feedback to the medic [22] based on a Wireless Body Area Network (WBAN) [23]. The watch has a size of 40 mm × 20 mm whose core component is a Tyndall 10 mm node and communicates to the sink node by Nordic nRF9e5 RF chipset via the 433 MHz radio band. Energy consumption issues still need to be solved.

A data-oriented system with reliable transmission and storage is essential for measurement of vital signs. A WSN-based platform in a homecare monitoring system is presented by Dobrescu et al. in 2008 to share expensive resources at a distance, increase diagnosis accuracy and treatment efficiency and save time to intervention [24]. They define a mobile node named Patient Wireless Node (PWN) and fixed node named Home Wireless Node (HWN), with the presented concepts still applicable today. With the technical limits of the time, an 8-bit microcontroller platform and General Packet Radio Service (GPRS) are employed in their test, so the performance of the platform is limited.

ZigBee defines a low-data-rate short-range wireless networking based on IEEE 802.15.4 [25,26], which is widely used in smart home systems for its low-cost implementation and low-power consumption [27,28]. A remote health monitoring system focusing on body temperature is proposed by Mansor et al. [29]. They employed the temperature sensor LM35 from Texas Instruments, sending the reading to Arduino by XBee, with the Arduino board receiving and decoding readings and sending results to the server by WLAN. Hand temperature measurements were considered in their experiments, with test results better than a commercial digital thermometer. One of the inconveniences is that they must hold the sensor in their hand for initial measurements. A more practical system by Kioumars and Tang adds the physiological signal of the heart rate, in which the sensor is wrapped around the wrist [30] and the Mean-Body Temperature (MBT) is calculated by Burton’s equation, with the heart rate measured by pulse oximetry. They found that motion, makeup and light can cause incorrectness in the measurement. A Long-term Wearable Vital Signs Monitoring System using BSN and ZigBee is proposed by Guo et al., covering other physiological signs such as ECG, SpO2 (Saturation of Arterial Oxygen) and systolic blood pressure [31]. The wearable device consists of two parts: a chest band connects with an ear-probe, as they are working on designing a delicate chest band for comfort and improving the system performance.

Another device implements an analogous function, but it is worn on the wrist and finger [32]. A mobile gateway for a ubiquitous health care system using Bluetooth to enhance the transmission capacity of ZigBee is presented by Laine et al. [33], improving the home care system and making it more flexible and feasible. Doukas and Maglogiannis brings IoT and Cloud Computing towards pervasive healthcare by reporting data to the cloud server through a lightweight REST-based API [34], thus completing the information reporting cycle from patient to clinician. Lloret J. et al. proposed a smart continuous eHealth monitoring system for chronic patients using 5G [35], they employed wearable devices, mobile phone and an intelligent DataBase for BigData, so heavy traffic needs extra bandwidth which is the advantage of 5G, but availability and affordability are current issues of 5G.

Some schemes based on pillow were proposed recently. An IoT-based smart pillow for sleep quality monitoring in Ambient Assisted Living (AAL) environments was presented [36], which integrates sensors of temperature, humidity, luminosity, sound, and vibration to a node of SparkFun ESP32 Thing, so environmental parameters can be adjusted automatically to meet need of qualified sleeping. The comfortability of the cushion and power consumption of node are trivial in lab but have to be addressed in product. An under pillow unperturbed sleep monitoring scheme identifies sleep stages S1–S4 with an accuracy of more than 60% [37], which is better than existing scheme based on hidden Markov model (HMM). It analyzes the pattern of heart rate variability (HRV) through obtained RR interval signal first, and employs ensemble empirical mode decomposition (EEMD) to eliminate errors from individual differences, then combines it with HMM to calculate corresponding stages, but the accuracy of this noninvasive monitoring system is still far from polysomnography (PSG). 

The proposed health sensing system is based on a smart pillow with temperature and humidity sensors. Besides keeping the comfortability of pillow in a noninvasive way, we concentrate on efficiency of energy, accuracy of measurement when designing and implementing the system. 

## 3. Health Sensing System

This section begins with a brief introduction of data flow in the system, then architecture and parts of the system which followed by details of the smart pillow, and finally how position and body temperature are extracted by Fuzzy Logic is presented.

**Figure 1:**
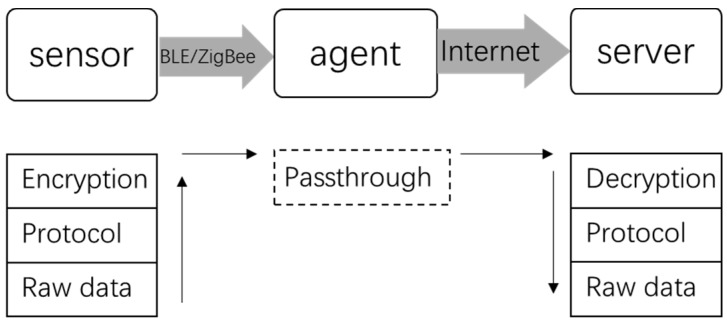
Data-flow block diagram of system.

The system consists of three parts: sensors, transport agent, and server. In order to upload data to the server, the Internet function has to be integrated into a host of sensors, such as wires, Wi-Fi, or 4G, which requires an AC power supply or a rechargeable lithium battery; although it can enlarge the host of sensors and in turn make it uncomfortable for the user. In contrast, BLE and ZigBee is designed with power saving capability, so they are perfect for short distance communication [38] and suitable for a host of sensors. As a result, the host of sensors is equipped with BLE/ZigBee, which is powered by a cell battery, and a transport agent is necessary in consideration of power consumption, which is equipped with both BLE/ZigBee and Internet capability. The data flow diagram of the system is shown in Figure 1.

Sensing data will pass through the agent from a host of sensors to the server, which means the data is not manipulated in the agent. On one end, the agent receives data from BLE/ZigBee, on the other end the agent sends data to the server by Internet. In the host of sensors, when the main chip gets raw data from Input/Output (IO) ports, it assembles the raw data according to an exclusive protocol first, then the assembled data is encrypted by specialized algorithms that is passed to the server by the agent. In the server, the reverse process is performed according to the specialized algorithm and exclusive protocol, then the final raw data will be analyzed to get results. As BLE or ZigBee is an open standard, encryption is significant for data security. An exclusive protocol is necessary to organize the sensing data with timestamping into a certain number of bytes to ensure data recovery on the server. If an encrypted algorithm or assembly protocol changes on one side, the other side should be modified accordingly.

### 3.1. Architecture of Current System

**Figure 2:**
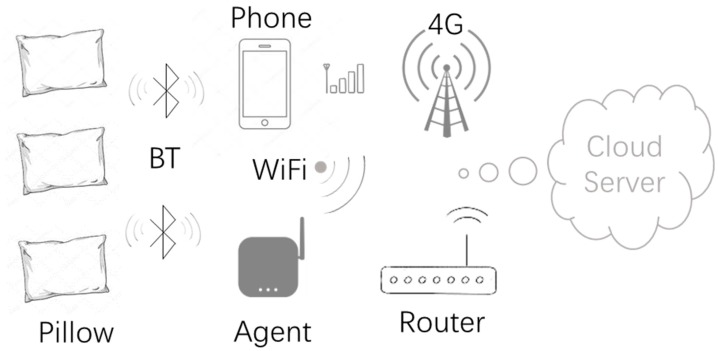
Current health system implementation.

The essential element of the current proposed health sensing system is the smart pillow, whose core component is a BLE single mode chip, which connects to three temperature sensors and one humidity sensor by input and output ports; communicating to the agent wirelessly by BLE. Sensory data is collected and pre-processed by the BLE chip and reported to a mobile phone or Wi-Fi agent by Bluetooth, then uploaded to the cloud server by Internet. Data is analyzed and health related features such as sweat, fever, insomnia etc. will be extracted in the server. A daily-based health report will be presented visually by webpage or returned to the mobile phone and visualized in a specified mobile application. Weekly, monthly, and annual health reports and suggestions are provided gradually in sequence by analyzing health related features based on known medical knowledge, or data is shared with professional medical organizations with consent of users, as it can provide an extra level of patient information and help in diagnosis and treatment. The architecture of the current system is displayed in Figure 2.

The pillow is data source of the system, which communicates via Bluetooth. Every pillow has a unique Bluetooth address and all pillows provide sensing data by an identical and specified service defined in BLE. The service makes pillows in the same class and address individualizes them. All the data needed is a timestamp to be more useful, but a single mode chip only maintains a timer by an internal Crystal oscillator; hence an external accurate time is synchronized when a connection is made to an agent.

The transport agent is equipped with both Bluetooth and Wi-Fi capability, the former collects data from the pillows, the latter reports data to the server, and requests accurate time from Internet to provide to the pillow. The connection between the pillow and agent is flexible, it could be from one agent to one pillow, or one agent to many pillows. Transport agent works in a polling mode, it scans for pillows by the specified service, setups a polling list, then connects to the pillows one by one, collects data and reports to the server with a unique Bluetooth address.

The server can distinguish pillows by their unique Bluetooth address. Results of data analysis in server cover three sections: statistics, inference and suggestion. Statistics is the simple and direct calculation or conclusion of original data, such as duration of sleeping, turns, room temperature and humidity, and sleeping position distribution, etc. The second part is challenging, as it includes body temperature and sweat sensing. It is difficult to measure accurate body temperature using a classic thermometer, so fuzzy logic is employed to infer body temperature based on head and environmental temperature. The other inferred element is sweat, which is a significant indicator for Chinese traditional medicine. The third section of the result is the recommendation of health, based on the conclusion from the first two sections and general medical knowledge.

The mobile phone is a perfect transport agent as most mobiles are equipped with Bluetooth, Wi-Fi and 4G, and it can even work without a server connection. The differences between a mobile phone and customized transport agent are listed in Table 1. Compared to a customized transport agent, the advantages of mobile phone are: it is more flexible as it has an inherent internet capability using 4G, it can analyze data locally and present visual results immediately after receiving data, but it has to be in the communication range of Bluetooth and the user takes the initiative to open a specified mobile application. On the contrary, a customized agent is plugged into a power socket and communicates to the pillow according to an automatic schedule, and all the received data is transported to the server transparently without analysis or presentation. A strong advantage for an agent is its ability of multi-connectivity. If more than one pillow is available in the Bluetooth connection range of agent, it can serve them all one-by-one.

### 3.2. Smart Pillow

**Figure 3:**
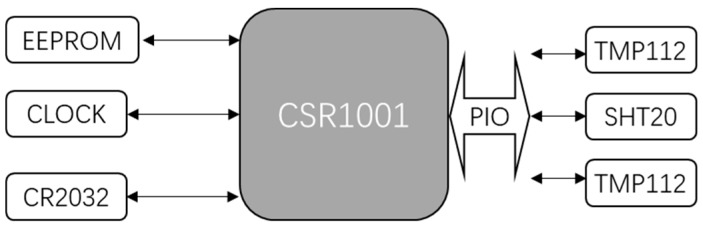
Hardware interface of sensors in smart pillow.

The block diagram of the host of sensors in the smart pillow is shown in Figure 3.

The host of pillow sensors is the CSR1001, which is a BLE single mode IC from CSR and powered by a cell battery CR2032. Two TMP112s (digital temperature sensors from Texas Instruments), and SHT20 (an IC combining humidity and temperature sensors from Sensirion) are connected to the PIO of CSR1001, so only three temperature sensors and one humidity sensor data will be collected from the pillow.

#### 3.2.1. Physical Structure

**Figure 4:**
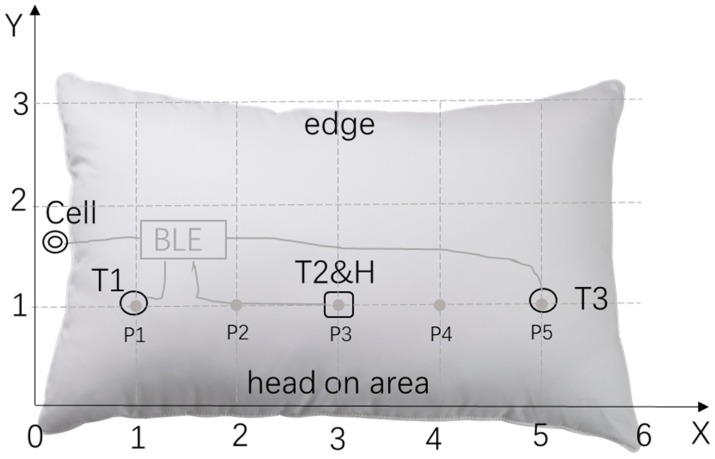
Physical structure of smart pillow as Cartesian coordinates.

Physical structure of smart pillow is presented as Cartesian coordinates in Figure 4. The pillow is set in a Cartesian coordinate system, one long edge of pillow is x-axis and one short edge is y-axis, so the bottom left corner of pillow is origin (0,0), and upper right corner is (6,3). Sensors are installed in the surface of pillow, they are shown in Figure 4 in black, so the first temperature sensor T1 should be installed at (1,1) which is marked as a circle, the third temperature sensor T3 at (5,1), the combination of second T2 and humidity sensor H1 in (3,1), which is marked as a square. They are wired to connectors in the BLE whose core is CSR1001. Wires and BLE core are hidden inside pillow, they are shown in Figure 4 in gray. A cell battery is exposed on the short edge of pillow, which is wired to BLE too. The long edge of pillow close to sensors, which *y* = 0, is the area where the head should always lay on. This is the recommended position of ideal installation, proportional relation of position of components should be always maintained in practice.

#### 3.2.2. Operational Principles

All the inferences are based on change of temperatures and differences between temperatures T1 to T3, except that humidity H1 is designed for sweat detection from the structure of pillow in Figure 4. The positions of the head-on the pillow are defined as 5 areas, their centers are marked as P1 to P5, which are P1(1,1,), P2(2,1), P3(3,1), P4(4,1), P5 (5,1). The sensor that is closest to head when sleeping is defined as main temperature (MT), and the sensor that is furthest to the head is defined as environmental temperature (ET), the left temperature sensor is defined as auxiliary temperature (AT); for instance, if head is on P1(1,1), T1 will be MT and T2 is AT, T3 is ET. It is not always the case that the roles of sensors are clear: for example, when head is on P4(4,1), only the fact that T1 is ET is clear, not T2 and T3, so their roles must be judged by the variety of temperature values.

Explanation of the mentioned statistical conclusions is straightforward. Duration of sleeping is available if timing of the head-on and head-off pillow are determined. Before the point of head-on, values of three temperature sensors are similar and stable, if any of them increases suddenly and dramatically, it is a sign of head-on. Head-off point can be judged in a similar but opposite way, if any of the temperature values drops and all of them become equal gradually, it is sign of the head-off. For room temperature and humidity, ET and H1 without head-on can provide related data; a turn in the pillow is defined as change of head position which can be inferred by dynamics of temperature data, and positions are described as sleeping position distribution. These indicators can be summarized as the habit of sleeping. The possible application of collected data is extensive, as only body temperature and sweat are extracted as significant medical indicators. There are more possibilities if large datasets of certain area of users in a longer period can be analyzed.

#### 3.2.3. Relationship of Position and Body Temperature

The first symptoms of many diseases start with a fever; hence an accurate body temperature is meaningful for the end user of the pillow. The position of the head is defined as 5 possibilities, from P1 to P5. From this definition, P1 and T1, P3 and T2, P5 and T3 at the same point respectively and P2 is in middle of T1 and T2, P4 is between T2 and T3. When extracting temperature, P1, P3 and P5 are in a similar situation that the head is right on the sensor, and P2, P4 is the other group, where the head is not exactly on a sensor, it is between two sensors. If the following two conditions are fulfilled, a body temperature is extracted:

- Head stays in a position, not less than 15 min; and
- The main temperature (MT) is available for capture.

For the first condition, when the head moves to a new position, the nearby temperature sensor needs a period of time for a stable value; 15 min is an experienced value as it relates to the material of a pillow case, performance of temperature sensor, and main temperature is the highest value that can be found at the point. The final core body temperature (CBT) is calculated by Equation (1).

CBT = T_main_ + T_material_ + T_adjust_ (1)

**Figure 5:**
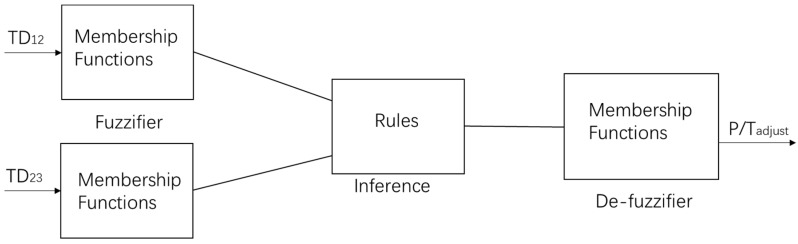
Data structure of fuzzy logic system for P/T_adjust._

In which T_main_ is the main temperature; T_material_ is material related value, and it is a verified value by repeated test on the specific material of pillow case, as the head does not directly meet the sensor. For P1, P3 and P5, only T_main_ + T_material_ as CBT is reasonable, but position P is defined as an area, the head is not exactly on point, it just is in an area whose center is the point, thus T_adjust_ is indispensable, it is a compensatory factor. In the case of P2 and P4, not a sensor is close to head as it is in P1 or P3, P5, so T_adjust_ should be higher. As a conclusion, position of head and extraction of body temperature are related closely. In the following diagram, inferring position and getting T_adjust_ by Fuzzy Logic are described in Figure 5.

Compared to classical logic in which the conclusion is true or false, Fuzzy Logic accepts partial true values. A Fuzzy Logic System (FLS) is nonlinear system that maps an input data set to a scalar output [39]. Two inputs are Temperature Difference (TD), TD_12_ and TD_23_ which TD_12_ = T1 − T2 and TD_23_ = T2 − T3; they are called Linguistic Variables and they will be fuzzified by their membership functions, then inferences will be made on a set of rules, after that all the inference results will be mapped to membership functions of P/T_adjust_; finally, a value of P/T_adjust_ is de-fuzzified as output. 

**Figure 6:**
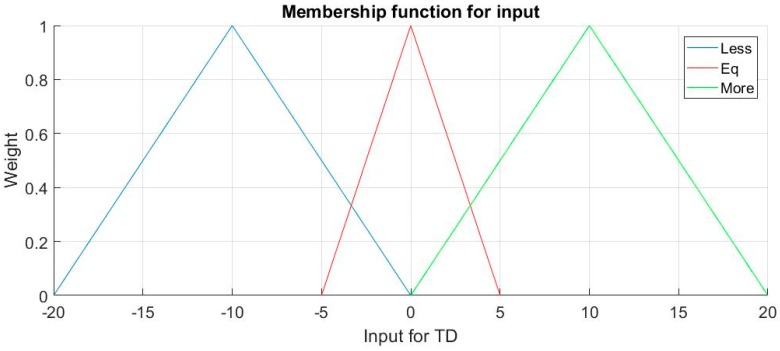
Membership functions μ(x) for input TD_12_ and TD_23._ TD: temperature difference.

TD_12_ and TD_23_ are employed instead of T1, T2, T3 because the judgment of position is based on differences of three sensors, not their values directly. The membership functions μ(x) for inputs are the same for TD_12_ and TD_23_, it is shown in Figure 6; the membership functions for output P and T_adjust_ are different, so they are discussed afterwards. 

Linguistic terms for TD is Term (td) = {Less, Equal, More}, for TD_12_, Less means T1 < T2 and More means T1 > T2 and so forth. The form of membership function is triangular, so for each term, in the exact middle, −10, 0, and 10, each membership function reaches its highest weight, 1, but parts of the functions are overlapped: for instance, td = 1 belongs to More and Equal at the same time, so both membership functions are effective. This is the essence of Fuzzy Logic, as td = 1 means no large difference of two temperatures, so it is reasonable to suit More and Equal.

**Figure 7:**
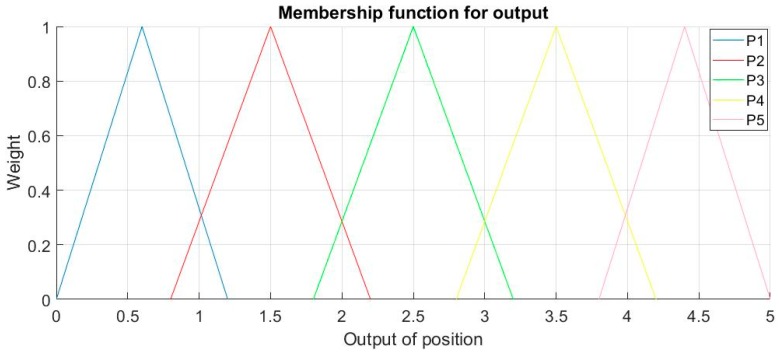
Membership functions for output P.

For output P, Linguistic terms Term (P) = {P1, P2, P3, P4, P5} and the output for P is a value in [0,5]. The membership functions for output P is shown in Figure 7.

The ultimate judgement of position will depend on {P1, P2, P3, P4, P5} = {[0,1), [1,2), [2,3), [3,4), [4,5)} to avoid any vagueness. In FLS, it depends on Rules to map inputs to output, the following rules are applied for output P in this FLS:

1. If (TD12 is Less) and (TD23 is Less) then (P is P5) (0.5)
2. If (TD12 is Less) and (TD23 is Less) then (P is P4) (0.5)
3. If (TD12 is Less) and (TD23 is Equal) then (P is P4) (1)
4. If (TD12 is Less) and (TD23 is More) then (P is P3) (1)
5. If (TD12 is Equal) and (TD23 is Less) then (P is P5) (1)
6. If (TD12 is Equal) and (TD23 is More) then (P is P2) (1)
7. If (TD12 is More) and (TD23 is Equal) then (P is P1) (1)
8. If (TD12 is More) and (TD23 is More) then (P is P2) (0.5)
9. If (TD12 is More) and (TD23 is More) then (P is P1) (0.5)

The format of the rule is observable, except the number after position, it is weight and can be interpreted as confidence or possibility.

The interpretations of those rules are listed:

- For rule 1 & 2, T1 < T2 < T3, position could be P4 or P5, head-on somewhere between P4 and P5, so the conditions are same, but conclusions are different, and weight is 0.5 for each.
- For rule 3, T1 < T2 = T3, head in the middle of T2 and T3, it is on P4.
- For rule 4, T1 < T2 > T3, head-on somewhere close to P3.
- For rule 5, T1 = T2 < T3, head-on P5.
- For rule 6, T1 = T2>T3, head-on P2.
- For rule 7, T1 > T2 = T3, head-on P1.
- For rule 8 and 9, T1 > T2 > T3, position could be P1 or P2, head-on somewhere between P1 and P2, so the conditions are same, but conclusions are different, and weight is just 0.5 for each.

There are two other possible conditions in theory, but not in the rules:

- T1 = T2 = T3, usually this is condition of empty pillow, no head-on so values of all sensors are equal, but from practical experience, if temperature of room is high enough to close to body temperature, it happens even head-on pillow, thus extreme weather is not considered.
- T1 > T2 < T3 is not in the rules because it cannot happen usually, only possible if user just turns around from one side of pillow to the other side, but the data from pillow is based on interval of 5 min, so momentary changes do not appear, and CBT is only extracted when head position is stable for no less than 15 min, so the option is improbable.

**Figure 8:**
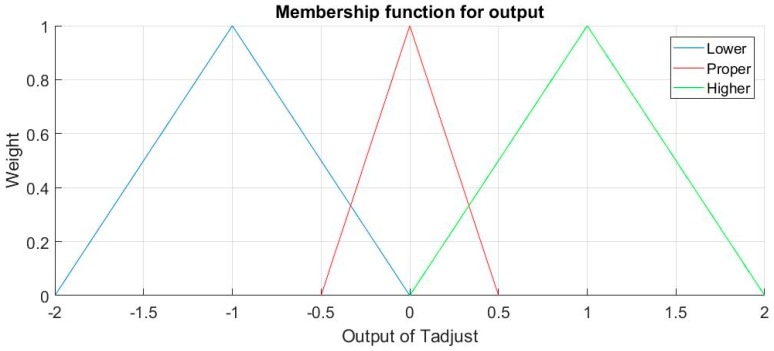
Membership functions for output T_a__djust._

For output Tadjust, Linguistic terms Term (Tadjust) = {Lower, Proper, Higher} and the adjustable range for Tadjust is [–2,2]. The terms are self-explanatory, for Lower the output of T_adjust_ will be negative, and positive for Higher. Membership function for output T_adjust_ is shown in Figure 8.

Rules designed for output T_adjust_ in this FLS are as stated:

1. If (TD12 is Less) and (TD23 is Less) then (adjust is Higher) (1)
2. If (TD12 is More) and (TD23 is Equal) then (adjust is Proper) (1)
3. If (TD12 is Equal) and (TD23 is More) then (adjust is Proper) (1)
4. If (TD12 is Less) and (TD23 is Equal) then (adjust is Proper) (1)
5. If (TD12 is Less) and (TD23 is More) then (adjust is Higher) (1)
6. If (TD12 is Equal) and (TD23 is Less) then (adjust is Proper) (1)
7. If (TD12 is More) and (TD23 is More) then (adjust is Higher) (1)
8. If (TD12 is Equal) and (TD23 is Equal) then (adjust is Lower) (1)

The principle for the rules is that if the head is on exactly defined positions, the CBT is proper so adjustment is not necessary, otherwise, adjustment should be positive, except that all three values of temperature are similar, which is mentioned before, heat cannot disappear in air as the environmental temperature is close to CBT, so the adjustment should be lower.

The rules are self-explanatory when presented in Table 2, in which all positions are observed in parenthesizes. 

In all rules stated above, “and” is used to combine TD12 and TD23, which still means an intersection in traditional logic, except that it is calculated by Min{μ_TD12_(x), μ_TD23_(x)}, which means the smaller value is employed.

The last step of FLS is de-fuzzification, which is finding a value for P/T_adjust_ in this case. By input rules, the appropriate membership function and the corresponding smaller weight are found. Then they are mapped to membership function of output, cut parts of membership function and form shapes, and the output value will be the center of gravity of the accumulated shape, it can be calculated by Equation (2).

(2) $$P/T_{\mathrm{adjust}}=\int_{\min}^{\max}t\mu \left(t\right)\mathrm{dt}/\int_{\min}^{\max}\mu \left(t\right)\mathrm{dt}$$

**Figure 9:**
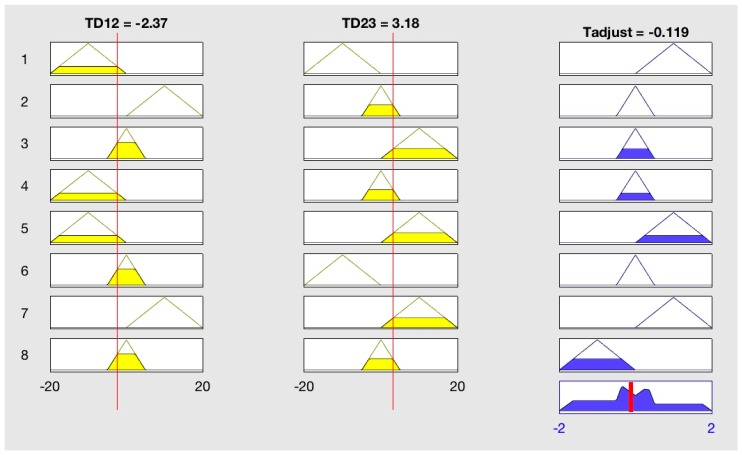
Visual process of input [−2.37, 3.18] in FLS for output T_adjust._

In which μ() is the membership function of output after accumulation, it is computable as it is combination of some triangles or trapezoids; t is value of P or T_adjust_, for P min is 0 and max is 5, and min is −2 and max is 2 for T_adjust_ in this case. The following Figure 9 is a visual process of the FLS when input of [TD12, TD23] is [−2.37, 3.18] for T_adjust_.

Rule 1, 6 are not met in this case as TD23 is not Less, neither rule 2, 7 as TD12 is not More, but both TD12 and TD23 are Equal as well according to their membership functions, so rule 3, 4, 5, 8 are all met. For rule 3, μTD12(−2.37) = 0.474 and μTD23(3.18) = 0.318, so 0.318 is transferred to output membership, making the proper membership function μ_Tadjust_(Proper) = 0.318. Explanations of other rules are similar, all the left shapes of rules accumulate to the final shape, then T_adjust_ is calculated by Equation (2), which is the gravitational center of the accumulated shape and marked with the red line in Figure 9.

Every time if a [T1, T2, T3] meets the standard of CBT, a [TD12, TD23] is calculated and fed to the FLS to output P/T_adjust_, then the real CBT is calculated by Equation (1).

## 4. Results

In this section, prototype of experimental trial pillow is presented first, then a daily sample is provided and interpreted, the sleeping patterns and reports are described in the end. 

**Figure 10:**
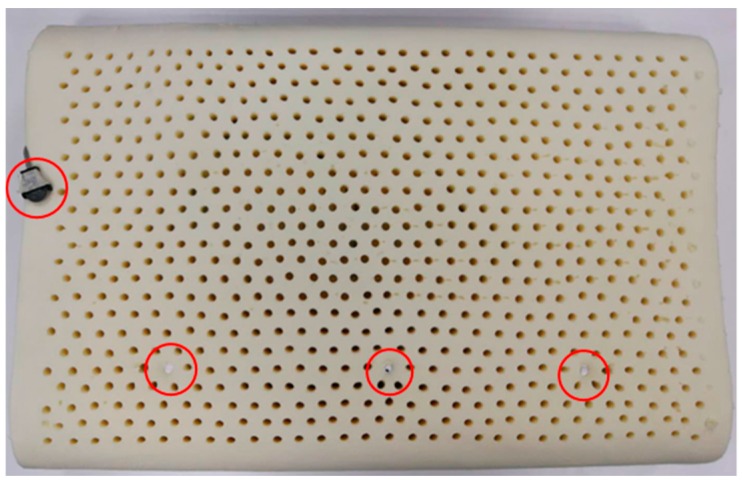
Prototype of experimental trial pillow.

The prototype of the trial pillow is displayed in Figure 10, which is a latex pillow with the advantages of comfort, support and easy maintenance. The master chip of BLE is CSR1001, which is a Bluetooth smart device from CSR, as it is hidden inside the middle of pillow and connected to 3 sensors and a cell battery. Three red circles at bottom of Figure 10 indicate where sensors are located. The left and right are TMP112s, which are digital temperature sensors from TI. The sensors are fixed to the surface of the pillow and their surface is coated with a white waterproof glue to prevent sweat from affecting their performance. The middle one is SHT20, which is a humidity and temperature sensor from Sensirion. Its surface is coated with white waterproof glue, except for the sensing window of humidity sensor, hence a small black point is visible from the figure. Inside the left red circle is a battery case for cell battery CR2032. Users are required to sleep on the side with the pre-positioned sensors.

To maintain the accuracy of the CBT, extensive experiments were carried out during the development stages. The experimental scenario applies 2 sets of sensing kits which is the combination of a CSR1001 connected with 3 sensors and a cell battery, shown in Figure 3. One kit is installed inside the pillow, shown in Figure 10, while the other is used to measure the real body temperature by fixing one of the temperature sensors in tester’s armpit. While testing, the tester’s head is on the pillow with the fixed temperature sensor under the armpit simultaneously; he/she then sleeps on a position steadily for 15 min, then moves to a different position on the pillow. After testing, data from both kits are extracted, CBT inferred by the data from the pillow is then compared to the direct result T_r_ from the armpit sensor. Then the algorithm is adjusted to meet the standard of |CBT − T_r_| ≤ 0.2 °C. Experiments were performed under an ambient environmental temperature of around 15 °C to 28 °C.

**Figure 11:**
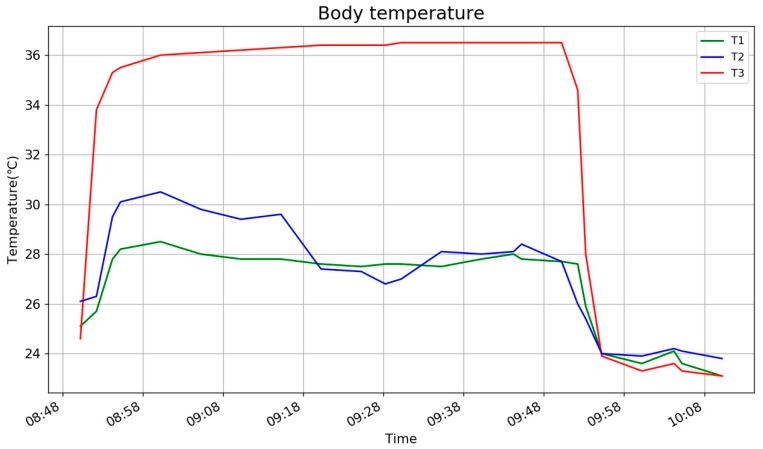
Measurement of real body temperature.

The measurement of real body temperature is shown in Figure 11. T3 is employed to detect the body temperature, as its value jumps to near 36 °C as soon as it is kept under the armpit but needs another 10–20 min to be close to the real body temperature. In our experiments, the maximum temperature value will be used as a reference for the pillow’s algorithm. In this way, the reference body temperature is measured at the same time, by the same type of device and under the same environmental conditions. However, it is noted that the measurements are taken in different parts of the body, of which it will be discussed further in the conclusion.

### 4.1. Interpretation of Whole Night Data

**Figure 12:**
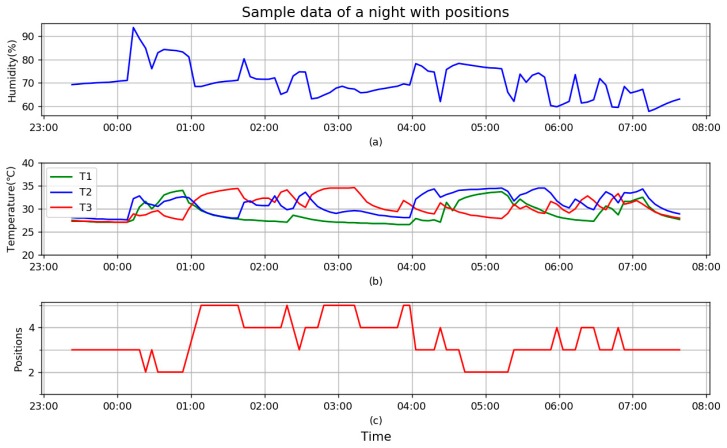
Sample data of sleep with (**a**) humidity H of a night, (**b**) T1, T2, and T3 of a night, (**c**) head-on positions of a night.

Data of a typical night sleep is presented in Figure 12.

Horizontal axis of the whole Figure 12 shows the time range (from 23:00 to 8:00 of next day), and the vertical axis of (a) shows humidity in percentage (%), at the beginning it is stable at about 70%, from about 00:08, it increases sharply up to 94%. It means head-on pillow from that time, then it changes a lot, up and down, as it implies that user rolls on the pillow, when head is on or close to the humidity sensor, it is up, otherwise down. At the end of the figure, it gradually returns to 65% in the morning after the user wakes up and leaves from the pillow.

Vertical axis of (b) is temperatures in Celsius (°C) of three temperature sensors. First, the blue line, which is T2, it changes almost synchronously in time with H in (a) although trends are not uniform all the time, because they are integrated in the same chip SHT20. For convenience of comparison, T1 is drawn in green and T3 in red. Like H at the beginning, all three values are 28 °C before head-on pillow, it is the room temperature, then values develop respectively when the user’s head is on and rolls on the pillow. Roles of temperature alternate with changes of head position. Values return to 28 °C in the morning after the user wakes up.

Vertical axis of (c) is head-on position, which is from 1 to 5, representing P1 to P5. According to the designed FIS, position of the head depends on differences of three temperature sensors: at the beginning when T2 increases sharply and faster than other sensors, it proves head-on T2/P3 which is in the middle of pillow. As P3 is the middle of pillow, position which is higher than 3 means head-on right side of pillow, lower means left. From (c) of the figure, the user is used to sleeping on the right side of pillow as positions of P4 and P5 is much more than P2. Number of turns can be counted from (c) if each change of position is treated as one turn.

The standard for extraction of body temperature is that main temperature lasts no less than 15 min. From (b) of the figure, at least three points are available, they are T3 at about 01:40, T3 at 03:10, and T2 at 05:15, they all meet the standard. Details will be described in the next figure.

### 4.2. Details of Typical Sleep Patterns

**Figure 13:**
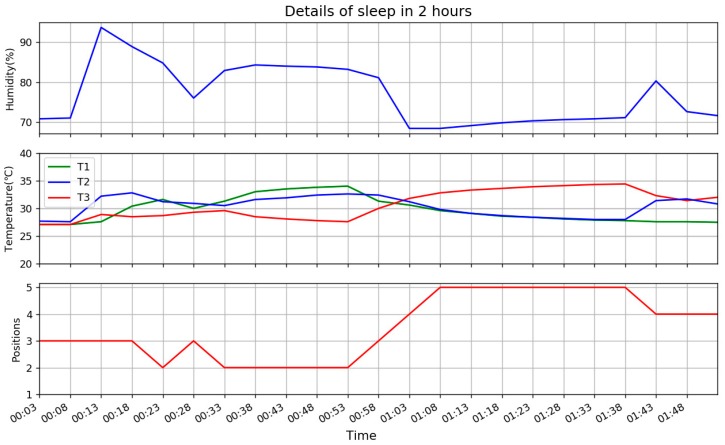
Extraction of body temperature data.

To describe more details of sleep, which includes extraction of body temperature, two hours of a night is presented in Figure 13.

As the interval of data from pillow is 5 min, time of data in the figure is from 00:03 to 01:53, every 5 min. Three parts of figure are the same as Figure 12, except more details in time. At the beginning of the figure, before 00:08, values of all three temperatures are similar and humidity is stable. It means no head is on the pillow, then suddenly T2 and H increase sharply and T1, T3 in different degrees of growth, because user goes to bed and head-on somewhere between P3 and P4 as T3 is faster than T1 in growth, then user moves slightly from somewhere between P3 and P4 to somewhere between P2 and P3 as T1 on the rise and T3 drops from 00:13. Then at 00:23 it is confirmed that head-on P2 as T2 drops but T1 and T3 are stable similarly. There is some confusion of the next data as the changes of values are not obvious, they are all close. But 5 min later at 00:33, T1 and T2 are up similarly and T3 drops which reclaims head-on P2. In the following 20 min, trend keeps stable, but it does not reach P1 as T1 and T2 keeps stable. After that the user turns, from left to right, so position changes from P2 to P3, P4 and finally P5 as T3 becomes leader and T1 and T2 drops synchronously. This lasts 30 min, then user turns slightly back to left, in P4 with T2 on the rise and T3 drops to meet T2 at 01:43.

### 4.3. Daily Report

From Figure 13, T1 at 00:53 and T3 at 01:38 meet the standard of body temperature, the 01:38 lasts longer, its values [T1, T2, T3] = [27.8, 28.0, 34.4], so [TD12, TD23] = [−0.2, −6.4], they are fed to designed FIS as input, output T_adjust_ = 0.0826, so CBT = 34.4 + 2 + 0.08 = 36.48 based on Equation (1). This is a typical position in P5, where T1 = T2 < T3, so the adjustment is small.

The other obvious body temperature point is found at 05:10 of Figure 12, it is a typical P2, T1 = T2 > T3, [T1, T2, T3] = [33.7, 34.5.0, 27.9], [TD12, TD23] = [−0.8, 6.6], T_adjust_ = 0.258 from FIS, it is greater than the last P5 adjustment, which is in accordance with theoretical analysis, so CBT = 34.5 + 2 + 0.258 = 36.76. Those two values are reasonable body temperatures.

Only one humidity sensor is installed in the middle of pillow, when the head is near P3, including P2, P3, and P4, H reflects the sweat of the head, otherwise it is just humidity of room. The observation of (a) and (c) proves this, when head is on P3, the humidity is high, if the head is far from P3, humidity drops to a local minimum that is the room humidity. At the beginning of Figure 12, humidity is up to 94%, which implies that user goes to bed without drying his/her hair, which Chinese medicine opposes strongly, but half an hour later, humidity is stable at about 84%, which means the user is in a condition of slight sweat. This will be included in the daily report.

As a conclusion, Table 3 is derived from the daily data as a report.

Bedtime is obvious from Figure 12, as all the values increase suddenly from flat as head-on pillow. Time of getting up is vague from Figure 12, usually the time that all values decrease or stabilizes and gradually gets close to room temperature is selected. In Figure 12, it is 07:08, so the total sleep time is available too. All the qualified body temperatures will be listed in report, in this case, the highest humidity is 94%, it will be reported that user sleeps without drying hair, and slightly sweats at night.

Turns in the report is counted on position changes, it is not real turns of sleeping as data from the pillow with an interval of 5 min. It means actions inside 5 min are ignored, but under the same standard, so the data is still meaningful for comparison.

The area of positions demonstrates which part of pillow user clings to during sleep. This can provide valuable reference as concerns arise on the relation of quality and sleep position.

## 5. Conclusions

The paper presents a smart pillow based on 3 temperature sensors and a humidity sensor, which is the core element of the Health Sensing System. Specifically, a report is provided daily by analyzing time series data from the pillow, which reflects the sleep status of a user faithfully like a camera, but it offers extra information such as body temperature and sweat. The daily summary is presented to user visually in a website or mobile application, numbers of one day are not of much significance, but comparison of daily data is more of value. From the weekly or monthly sleep data, the user can observe the dynamic status of sleep day-by-day: improvement, worse, fever, sweat, then take appropriate actions initially to concentrate on his/her sleep quality, such as sleeping earlier, or consulting professional help. This is just the comparison of an individual—if statistical data of all users can be analyzed, users can find their sleep levels in overall. Once pertinent questions can be asked by an individual when comparing with a larger dataset, this will ultimately help the patient improve their sleep quality and health.

An obvious issue of the pillow is that data is only reported every 5 min. All the details inside the 5 min are lost. First, this is a compromise for the energy efficiency of BLE. As described before, the core of pillow, which is a CSR1001 BLE chip, is powered by the cell battery CR2032. Compared to ubiquitous lithium battery, it has lower energy capacity and is non-rechargeable, but it is safer, smaller, and easier to be accepted by the user of pillow. The other question is how long the appropriate interval is. From the viewpoint of provided report, accuracy of turns is affected seriously by interval, all other items are less dependent. For Bedtime and Getting Up, the largest possible error is 5 min, which is trivial compared with total sleep time. A larger interval means less data and lower power consumption, so therefore longer battery life. In our extensive experiments, a cell battery can maintain normal function for 3 months of general use. It is acceptable and reasonable for the user to change the battery about once a season.

To the best of my knowledge, our smart pillow is the only mass-produced and temperature-related smart pillow on the market, the other two temperature-related pillows are neck pillow [15] and cushion [36], they are just tested in labs. Comparison of them are presented in Table 4.

Accuracy of neck pillow and cushion is higher as the former employing DHF and Double Sensors, the latter installing sensor on the outside for just ET not CBT, the cost of both are higher as they use more sensors, as a result their energy consumption are higher too, and sleeping on a cushion is more comfortable than with neck pillow. 

From massive experiments of our pillow, the inferred normal CBT is very closed to normal adult temperature based on the proposed Fuzzy Logic system. It is not compared with body temperature measured by clinical thermometer as body temperature is deeply related to time, position, way and instrument of measurement. So the accuracy of CBT is less weighted as change of CBT. As a commercial product, comfortability and energy efficiency is our advantages.

## 6. Further Work

Additionally, the smart pillow could employ energy scavenging techniques typically applied for wireless sensor networks and the Internet of Things. While it would make the pillow more complex to manufacture, the advantage is that the pillow would have more energy reserve to support additional sensors, or last longer between battery replacements. Pressure-based or heat-differential energy capture methods can be employed to add to the total energy reserve of the pillow. Therefore, a balance can be established to make a logical trade-off between utility and comfort, so that a more comprehensive pillow with a longer energy life can be made to support more sensors for specific patients in mind, or a simpler constructed pillow for general use.

There are various ways to measure body temperature clinically, and accuracy is different. Accuracy of body temperature obtained from the smart pillow is closely related to environment temperature and the material of the pillowcase according to our experiments. The essential problem is that heat dissipation is limited by them. If environment temperature is high enough to be close to body temperature, it will be difficult for heat to escape from the pillow, and as a result, the value of temperature sensor changes slowly, which is harmful to our algorithm as it deeply depends on difference of temperature. Some materials of pillowcase also cause obstacles to heat dissipation, so usually the smart pillow is provided with a case and other cases are not recommended, then the value of T_material_ is accurate in our algorithm, but with massive data, an appropriate T_material_ + T_adjust_ could be learned based on normal body temperature.

Further work needs to be done to obtain core body temperature from the pillow and a reference point that is as close as possible to both source points. The current experiments collected the body temperature from a different source point to the pillow, due to uncomfortable nature of placing a thermometer near the user’s head while it is also on the smart pillow. Special techniques will need to be developed to place the thermometer in such a way to reduce the incidence of user discomfort, otherwise the results will be affected by excessive head turns or other unanticipated body movements.

As a health sensing system, body temperature and sweat measurement are only the start of development; if some other clinical indicators such as blood pressure and heart rate can be integrated into the pillow or system it will make the system more useful. Theoretically, it is possible to consider a photo-plethysmogram (PPG) [40,41], as it is extensively applied in smart devices for measurements, but it is an optical technique that requires a camera and light source such as an LED flash to operate effectively. Integrating more components will make the pillow more complex to manufacture, and worsen comfort and increase battery consumption, so adding a smart device with blood pressure and heart rate detector could be an optimal decision.

The prospect of big-data on sleep and health is promising evolution to the path of connected home medicine. Not only can it discover hidden disease in advance in a non-intrusive, natural, and unconscious way, it can open new developments to determine medical trends among patients for better medical diagnosis—hence the necessity for the design of a smart pillow.

## Figures and Tables

**Table 1:** Comparison of using mobile phone versus customized transport agent.

Items/Devices	Mobile Phone	Customized Agent
Data process	Analysis/passthrough	Passthrough
Internet access	4G/Wi-Fi	Wire/Wi-Fi
Presentation	App	No screen
Time to connect	Any time	Scheduled
Connect to pillow	Manual	Auto
Multi-connection	One pillow	Multi-pillows
Accurate timer	Inherent	Time sync from server
Flexibility	Flexible	Fixed

**Table 2:** Matrix of rules for fuzzy logic system.

TD12/TD23	Less	Equal	More
Less	Higher (P4/P5)	Proper (P4)	Higher (P3)
Equal	Proper (P5)	Lower	Proper (P2)
More	None	Proper (P1)	Higher (P1/P2)

**Table 3:** Daily report for sample data.

Item	Value
Bedtime	00:08
Get up	07:08
Sleep time	7 h
Body Temperature	36.5/36.8
Sweat	94%
Turns	37
Left/Middle/Right	14/30/40

**Table 4:** Comparison of three temperature-related pillows. CBT: core body temperature; ET: environmental temperature.

Name	Accuracy	Comfortability	Energy Efficiency	Cost	Purpose
Neck pillow	High	Low	Low	High	CBT
Cushion	High	Mild	Low	High	ET
Smart pillow	Mild	High	High	Low	CBT
